# Broad Reach and Targeted Recruitment Using Facebook for an Online Survey of Young Adult Substance Use

**DOI:** 10.2196/jmir.1878

**Published:** 2012-02-23

**Authors:** Danielle E Ramo, Judith J Prochaska

**Affiliations:** ^1^Department of PsychiatryUniversity of California, San FranciscoSan Francisco, CAUnited States

**Keywords:** social media, Facebook, participant recruitment, young adult, tobacco

## Abstract

**Background:**

Studies of tobacco use and other health behaviors have reported great challenges in recruiting young adults. Social media is widely used by young adults in the United States and represents a potentially fast, affordable method of recruiting study participants for survey research.

**Objective:**

The present study examined Facebook as a mechanism to reach and survey young adults about tobacco and other substance use.

**Methods:**

Participants were cigarette users, age 18-25 years old, living throughout the United States and recruited through Facebook to complete a survey about tobacco and other substance use. Paid advertising using Facebook’s Ad program over 13 months from 2010 Feb 28 to 2011 Apr 4 targeted by age (18-25), location (United States or California), language (English), and tobacco- and/or marijuana-related keywords. Facebook approved all ads.

**Results:**

The campaign used 20 ads, which generated 28,683,151 impressions, yielding 14,808 clicks (0.7% of targeted Facebook members), at an overall cost of $6,628.24. The average cost per click on an ad was $0.45. The success of individual ads varied widely. There was a rise in both clicks and impressions as the campaign grew. However, the peak for clicks was 3 months before the peak for ad impressions. Of the 69,937,080 accounts for those age 18-25 in the United States, Facebook estimated that 2.8% (n = 1,980,240) were reached through tobacco and marijuana keywords. Our campaign yielded 5237 signed consents (35.4% of clicks), of which 3093 (59%) met criteria, and 1548 (50% of those who met criteria) completed the survey. The final cost per valid completed survey was $4.28. The majority of completed surveys came from whites (69%) and males (72%). The sample averaged 8.9 cigarettes per day (SD 7.5), 3.8 years of smoking (SD 2.9), with a median of 1 lifetime quit attempts; 48% did not intend to quit smoking in the next 6 months.

**Conclusions:**

Despite wide variety in the success of individual ads and potential concerns about sample representativeness, Facebook was a useful, cost-effective recruitment source for young-adult smokers to complete a survey about the use of tobacco and other substances. The current findings support Facebook as a viable recruitment option for assessment of health behavior in young adults.

## Introduction

Studies of tobacco use and other health behaviors have reported great challenges in recruiting young adults [1,2]. The Internet, increasingly used as a method to target and survey individuals about health-risk behaviors, may be a useful tool for reaching young adults. Compared to face-to-face interviews, Internet-based surveys can reach more potential respondents; enable inclusion of low-incidence or “hidden” population groups; enable rapid, convenient input by respondents; and reduce bias in response to sensitive, potentially stigmatizing topics [3-7]. A recent telephone survey of young adults age 18-29 in the United States indicates that almost all (93%) use the Internet [8]. Further, over the past decade, young adults have remained the age group most likely to go online.

Studies of Internet-based tobacco-cessation treatment have demonstrated high enrollment among general-aged adult participants through advertisements on Google or other search engines [9]. An intervention for smokeless tobacco (Chewfree.com) used advertisements on Google.com and generated 9155 clicks and 511 intervention participants at a cost of $6.70 per participant; advertisements on other search engines generated 363 participants (mean age, 34.5 years) in 15 months [10]. In another online smoking-cessation intervention (Quitnet [11]) advertisements on Google resulted in 28,296 clicks, producing 5557 eligible participants. Of these, 1489 gave informed consent and 764 (mean age 35.1 years) completed a baseline assessment in 6 weeks. In our previous work using the Internet to recruit young-adult smokers in survey research, advertisements across the Web yielded the largest proportion of recruited participants and completed surveys overall; however, Craigslist and an online sampling company were more successful at targeting young-adult smokers who went on to complete the survey and ultimately were the more cost-effective methods compared to Internet advertisements [12]. 

Social media is widely used by young adults in the United States. Nearly three quarters (72%) of online 18-29-year-olds use social-networking websites, with 45% doing so on a typical day [8]. Facebook, the largest social-networking website and second most popular website in the United States after Google [13], has more than 500 million users worldwide, half of whom use the site daily [14]. Facebook represents a potentially fast and affordable method of recruiting study participants for survey research, especially young adults who use the site in large numbers and on a frequent basis. Given the increasing interest in using social media for recruiting participants to research, the current study reports on the success of an ad campaign on Facebook, the leader in this space. We believe this is the first study to examine Facebook as a mechanism to reach and survey young adults about health behavior.

## Methods

### Participants

The target population was young-adult cigarette users, age 18-25 years, living throughout the United States. Individuals had to be English literate and smoke at least one cigarette in the past 30 days to be eligible for participation.

### Facebook Recruitment Campaign

To reach young adults who had smoked recently, we paid for advertising using Facebook’s Advertising (Ad) program over 13 months from 2010 Feb 28 to 2011 Apr 4. Our campaign involved creating advertisements that appeared on the pages of our target audience meeting the criteria of age (18-25), location (United States or California), language (English), and tobacco- and/or marijuana-related keywords that appeared in their Facebook profiles through listed interests, activities, education and job titles, pages they like, or groups to which they belong (eg, “cigarette,” “nicotine,” “blunt,” “420”). At the time of this campaign, this was the only way Facebook ad could be targeted (ie, there was no way to target keywords to other areas of the Facebook profile). Facebook had to approve all ads based on the company’s guidelines [15]. Only one ad type was available to advertisers at the time the campaign was launched. Ads included a short (eg, 2-word) headline, a picture, and a link to the study’s survey website per Facebook’s advertising size and word-count specifications. Facebook rejected an ad that targeted both tobacco and marijuana users through pictures. Therefore that ad made no impressions. We incurred a charge every time a user clicked on one of our ads.

On a daily basis, we could specify a spending limit for each ad and for the entire campaign. We, like other advertisers, could then specify the maximum amount we would be willing to pay for an ad (a “bid”). Then auctions determined the likelihood a given ad would be shown on pages of the target audience. Selection criteria included bid (the amount an advertiser is willing to pay), quality of an ad (including feedback an ad has received from users), and past performance [16]. For a given ad, Facebook suggests a “bid range” based on how much other advertisers would be willing to pay to reach the same target audience. This range can change over time based on both the ad space (other ads in the pool of ads) or an ad’s performance. Our bids fluctuated over time in line with the bid range for a given ad. Facebook reports statistics on bids, impressions, clicks, and dollars spent on all ads in a campaign. Impressions are defined as a single time an ad is shown to a user, regardless of whether the user clicks on the ad. Clicks are when a user clicks a link in an ad. We used the Facebook-provided statistics indicating the success of each advertisement we ran and changed or stopped ads that were unsuccessful (ie, they rarely appeared, they received too few clicks, or they were too expensive). As such, we explored various picture and text options and determined the most successful ads based on impressions, clicks, and costs. Facebook, which normally restricts the reporting of its data, gave us permission, conveyed to our university’s legal counsel, to publish these statistics.

### Study Procedures

The Institutional Review Board approved study procedures, described in detail previously. Participants provided informed consent [12,17]. The online survey included basic demographics and a series of measures of smoking and other substance-use behaviors and thoughts about use. A Smoking Questionnaire assessed participants’ years of smoking, prior quit attempts (lifetime and past year), and longest period of abstinence in a prior quit attempt [18]. The Smoking Stages of Change Questionnaire [19] assessed motivation to quit, categorizing smokers into 1 of 3 preaction stages of change: precontemplation: no intention to quit within the next 6 months; contemplation: intention to quit within the next 6 months but no 24-hour quit attempt in the past year; preparation: intention to quit within the next month and a 24-hour quit attempt in the past year. The Thoughts about Abstinence form [20] assessed desire to quit, anticipated success with quitting, and perceived difficulty with abstinence (each rated on a scale of 1 to 10). Participants were required to answer all questions before they could continue to the next page of the survey and could quit the survey at any time. Computer Internet Protocol (IP) addresses were tracked and multiple entries were not accepted from the same computer. Data were deemed invalid and excluded from analyses if (1) there was a discrepancy in data from duplicate questions (eg, date of birth; *n* = 215), (2) respondents reported the same contact email address across multiple survey entries (*n* = 50), or (3) the data were clearly invalid (eg, every entry was the same across the entire survey; *n* = 28). At the end of the recruitment period, each completed survey entry associated with a valid email address was entered into a drawing to win a $400 gift certificate to Apple stores or a $25 gift certificate to a national or online store.

## Results

### Advertising Campaign

During the 13-month campaign, our ads made 28,683,151 impressions, yielding 14,808 clicks, at an overall cost of $6,628.24. The average cost per click on an ad was $0.45. Twenty different ads were run. Of those, 14 were targeted throughout the United States and 6 specifically to California; 7 asked participants if they “ever smoke,” 7 asked if they “ever smoked cigarettes,” and 6 asked if they “smoked recently;” 8 had a cartoon picture, including lit cigarettes (n = 3) and a cigarette pack (n = 5); and 12 had a realistic picture, including a lit cigarette (n = 3), a cigarette pack (n = 3), multiple cigarettes (n = 3), and a person smoking a cigarette (n = 3). 

**Figure 1:**
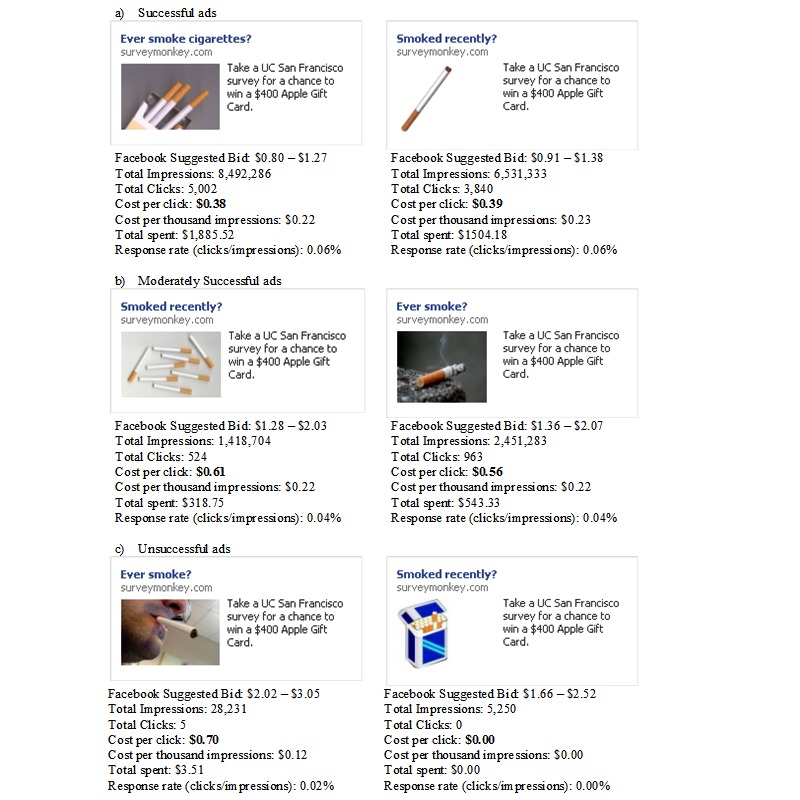
Examples of two successful (1a), moderately successful (1b), and unsuccessful (1c) advertisements from the Facebook campaign based on ad statistics. Reported Facebook-suggested bids are from the last day of the campaign.

The success of individual ads varied widely. Figure 1 illustrates, as an example, 2 sets of 3 ads deemed highly successful, moderately successful, or unsuccessful based on their campaign statistics. The most successful ad in our campaign had over 8 million impressions, which yielded over 5000 clicks, and cost $0.38 per click (Figure 1a). Other ads varied in the number of clicks and costs per click (eg, Figure 1b). Some ads made impressions but were not clicked on at all. For these we did not incur any charges (Figure 1c).

**Figure 2:**
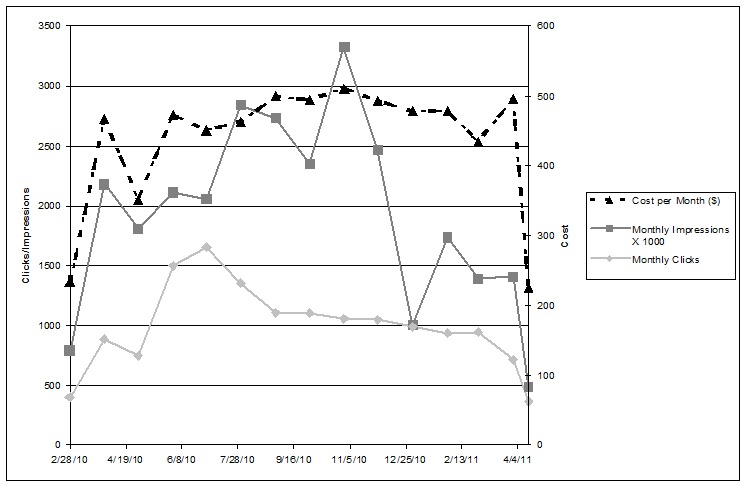
Total monthly impressions and clicks (left axis) compared to average monthly dollars spent (right axis) across the 13-month Facebook recruitment campaign.

Figure 2 presents the rate of impressions and clicks compared to campaign costs for the entire campaign over 13 months. There was a rise in both clicks and impressions as the campaign grew. However, the peak for clicks was in the summer of 2010, which was 3 months before the peak for ad impressions (October 2010). There was a large drop-off in impressions between October 2010 and January 2011, without a corresponding shift in clicks or costs, highlighting that the clicks were coming from a few consistently successful ads throughout that period.

### Recruitment Results

**Figure 3:**
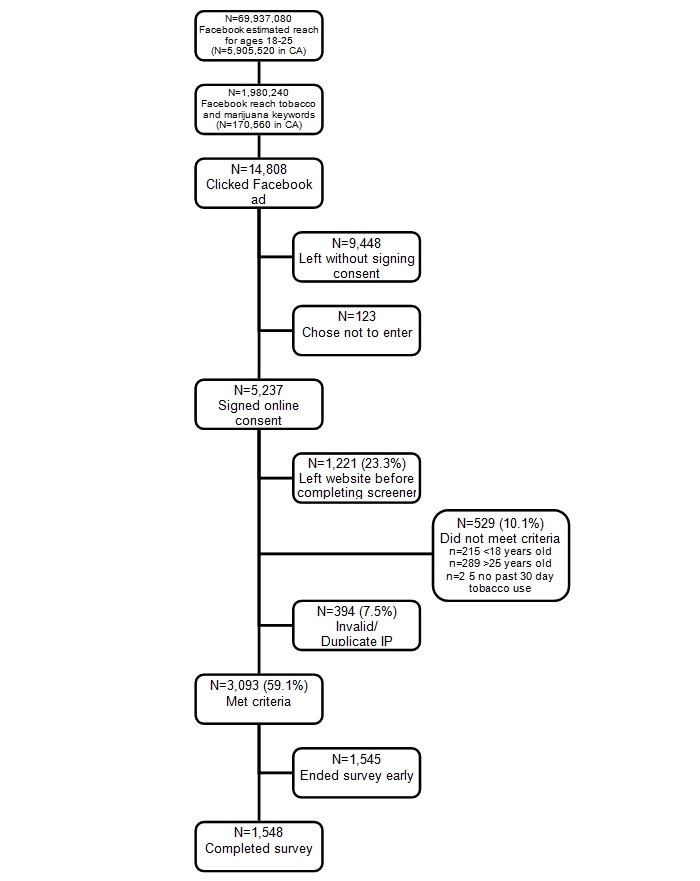
Facebook ad campaign reach and recruitment process.

Figure 3 summarizes the numbers of potential Facebook accounts reached through various target characteristics, the clicks our ads received, and the sample who reached and completed our survey. Of the 69,937,080 Facebook accounts registered to individuals age 18-25 in the United States, 2.8% (n = 1,980,240) had profiles with tobacco- and marijuana-related keywords (our target population). Our campaign received 14,808 clicks (0.7% of potential accounts reached through the campaign), which yielded 5237 signed consents, of which 3093 (n = 59%) met study-inclusion criteria, and 1548 (50% of those who met criteria) completed the survey. The final cost per valid, completed survey was $4.28.

### Participant Characteristics

Table 1 presents sociodemographic and tobacco-use characteristics of the sample that completed the survey. The majority of participants were male and white. All four US census regions were represented, with the Northeast having the lowest representation (20.2%). The sample averaged 8.9 cigarettes per day (SD 7.5) and 3.8 years of smoking (SD 2.9). Sixty-three percent reported a lifetime quit attempt of at least 24-hours’ duration. At the time of survey completion, 48% were not intending to quit smoking in the next 6 months (ie, precontemplation stage of change); 30% were contemplating quitting in the next 6 months, but not in the next 30 days (ie, contemplation); and 23% were preparing to quit in the next 30 days and reported a quit attempt in the past year (ie, preparation).

**Table 1:** Demographic and smoking characteristics of young adults who completed the survey (N = 1548)

General characteristics		% or mean (SD)
**Gender, %**		
	Female	30.6
	Male	68.9
	Transgender	0.5
**Age in years, mean (SD)**		20.3 (2.0)
**Race/ethnicity, %**		
	African-American/black	2.5
	Asian/Pacific Islander	3.6
	White	71.7
	Hispanic/Latino	6.1
	Other	16.1
**Employment status, %**		
	Full-time	28.7
	Part-time	17.0
	Unemployed/homemaker	24.0
	Student	30.3
**Years of education, mean (SD)**		13.0 (1.9)
**Annual family income, %**		
	Less than $20,000	25.3
	$21,000-$40,000	20.0
	$41,000-$60,000	14.8
	$61,000-$80,000	11.2
	$81,000-$100,000	9.4
	Over $100,000	19.4
**Subjective social status, mean (SD)**		5.8 (1.9)
**Region, % **		
	Northeast	20.2
	Midwest	26.9
	South	27.1
	West	25.8
**Number of cigarettes smoked per day, mean (SD)**		8.9 (7.5)
**Years smoking, mean (SD)**		3.8 (2.9)
	Prior quit attempts in lifetime, mean (SD)	8.5 (26.6)
	Quit attempts in past year: median (interquartile range)	1.0 (3)
**Stage of change, %**		
	Precontemplation	47.5
	Contemplation	29.6
	Preparation	22.9
**Desire to quit, mean (SD)**		5.3 (3.0)
**Expected success, mean (SD)**		6.0 (2.9)
**Expected difficulty, mean (SD)**		6.5 (2.8)

## Discussion

Overall, Facebook was a successful recruitment source for young-adult smokers to complete a survey about tobacco and other substance use. At $4.28 per completed survey, this method proved much more affordable than other methods we used in previous survey research with this population, especially given the minimal staff time to design and monitor the campaign. In our prior work, the cost per completed survey was $42.77 for an Internet marketing company to place ads throughout the web and $19.24 for a survey sampling company to email survey announcements to young adults [12]. Traditional recruitment mechanisms, such as newspaper or radio advertising or hired survey sampling companies, tend to be far more expensive and take more time for participant screening. Other Internet-based recruitment mechanisms that have proven successful at recruiting for smoking-cessation interventions, such as Google’s AdWords program [9,10], are not as easy to use for targeting a specific demographic population. As a result, such mechanisms require users to screen participants more thoroughly and to validate data generated through these recruitment methods.

Facebook is a useful mechanism for recruiting young research participants in a field that has shown past challenges. Nearly half (48%) of those completing the survey were unmotivated to quit smoking within the next 6 months. Smokers unmotivated to quit may be particularly challenging to recruit into face-to-face research studies, further highlighting the value of online methods. The current findings indicate that Facebook, compared to other mechanisms, may be particularly effective at recruiting smokers unmotivated to quit in the near future as the proportion was higher than that in our prior research of online recruitment of young-adult smokers [12]. Young adults are conducting daily communications at an increasing rate via mobile methods, rather than by face-to-face encounters or by telephone at home, making traditional methods of recruitment and assessment increasingly obsolete [8]. Researchers desiring to understand health behavior of young adults (especially stigmatized behavior) should consider social media as a viable option for recruitment.

The campaign’s success was predicated upon Facebook’s approval of each advertisement. The advertising policy strictly forbids the sale of or reference to tobacco products as well as mention of illegal activity [15]. To obtain Facebook approval of our tobacco ads, we provided evidence that we represented an academic research study. However, an ad with a picture of a marijuana leaf on it was not approved. Facebook’s wide reach makes it a useful way to target hard-to-reach populations; however, to adhere to Facebook’s guidelines, studies dealing with illegal activity, such as underage alcohol use or illicit drug use, may be required to use more neutral words and pictures, and thus may reach a wider population than their intended subject pool.

A limitation to recruitment through Facebook is that the representativeness of the sample cannot be fully determined. At the time of recruitment, Facebook reported the number of total Facebook accounts potentially targeted by any given set of demographic characteristics or keywords; however, there was no ability to compare this larger population to the eventual sample generated through an ad campaign. At the time the campaign was implemented, there was also no way to determine how many individuals within the population were exposed to at least one advertisement. It is possible that Facebook will choose to make this information available but it is at their discretion. The data that Facebook did provide for each ad (eg, impressions, clicks, costs) made it simple to continuously evaluate and optimize our campaign.

The research presented here used a cross-sectional survey design. There is limited knowledge about Facebook’s success at reaching and retaining young adults in longitudinal treatment-outcome studies or clinical trials. Given the high proportion of recruited smokers unmotivated to quit, tobacco-treatment models that do not require immediate cessation (eg, stage-tailored interventions, smoking reduction) may be particularly appropriate, although this should be confirmed. As a common method of communication for young people all over the world, social media represents a useful strategy that can be leveraged for research to find and engage potentially hard-to-reach populations.
